# Syphilis

**Published:** 1898-04

**Authors:** J. T. Benbrook

**Affiliations:** Rockwall, Texas


TEXAS MEDICAL JOURNAL.

ESTABLISHED JULY, 1885.

PUBLISHED MONTHLY.—SUBSCRIPTION $2.00 A YEAR.

VOL. XIII. AUSTIN, APRIL, 1898. No. 10.

### Original Contributions.

For the Texas Medical Journal.

### SYPHILIS.

BY J. T. BENBROOK, ROCKWALL, TEXAS.

Buret, one of France's most eminent syphilographers, says, that syphilis is as old as prostitution itself, that it was conceived, born and nourished in the hot-bed of prostitution, and that by prostitution and debauchery it has been nourished, propagated and kept alive through all the ages down to the present time. Some other writer has said that prostitution commenced with our great grandmother, Eve, who, by her cunning, seduced poor, helpless Adam in the Garden of Eden.

Now, I don't mean to insinuate that Eve had syphilis, or that Adam ever had syphilis, for I don't believe that a woman can have syphilis—barring heredity—until she has a chance to catch it, and we have most positive evidence that up to the time of that garden scene, the virtue of Miss Eve had never been questioned; nor do I believe that a man can give a woman a case of syphilis when the man himself is not infected with the virus, and has no opportunity of beiug inoculated; therefore, we must conclude, if we accept the teaching of divine history, that Adam's reputation for chastity was just as good as was that of his companion—Eve; neither having syphilis, one could not transmit it to the other.

Prefacing my remarks on this subject, I have deemed it expedient to step aside and exonerate our parents in the Garden of Eden from having anything to do with visiting this curse upon their progeny.

Syphilographers disagree as to the antiquity and the origin of this malady. Passing back a few decades, it was pretty well conceded by leading authorities of the day that syphilis was introduced into the old world by the Spaniards on their first return voyage with Columbus, after having discovered America, thus giving America the uncoveted honor (?) of being the legitimate parent of syphilis. More recent and more thorough investigation, however, has conclusively proven the existence of syphilis in prehistoric ages. A careful study of certain human bones exhumed in various parts of the earth, exhibit lesions identical in character to those recognized in bones where there is no question as to the syphilitic nature of the process. It is an accepted theory of some syphilographers that Pharaoh, with the whole Egyptian court, was infected by the beautiful Jewess, Sarah, Abraham having carried her down into Egypt on account of the famine in Bethel. Pharaoh was struck with her beauty, and simply took charge of her to gratify his own lust. But "every sweet has its bitter." The king contracted syphilis, transmitted it to his wives and housemaids, and through them to the whole court. This story dates back nearly four thousand years. Following biblical history along, nine hundred years later we come to the little episode between King David and Bath-Sheba, and the tragic taking off of Uriah. Bath-Sheba was syphilitic. David's sin had its swift retribution, and in his psalms he is made to cry, "My ulcers are putrid and corrupt on account of my folly." "He wept sometimes for his sin, but always kept his mistress."

Just how the first syphilitic germ was born, and in the anatomy of just what particular person or nationality of people it first thrived, multiplied and extended to others of his race, is not a question in which we are so much interested as we are in the question, "How are we to meet and successfully combat this disease?" There is no clime or nationality under the sun that has not felt its baneful influences; it is no respecter of persons or personal rights; it invades alike the peasant in his hovel and the prince in his palace; it takes the innocent babe from the cradle by the shortest route to the cemetery; it takes hold of the adult youth of vigor and promise and makes him a weakly, rickety, helpless imbecile; it visits the sins of the licentious and debauched upon the heads of the innocent and the pure. It is a disease to be loathed above every other disease known to modern pathology because it is of bastard parentage, and but for the sin of prostitution one half century would blot its existence from the world.

We care not to theorize further as to how long it has been in the world, how it got here or who first had it; we want to meet conditions; not theories.

Syphilis is an infectious disorder, both acquired and transmitted by inheritance, chronic in course and manifesting in successive order specific symptoms which may be declared in one organ of the body, or simultaneously or successively in several. Its specific pathology is due to a toxic effect, by the invasion of the tissues of the body of a micro-organism whose identity and relation are not, as yet, satisfactorily understood. It is characterized by three distinct periods or stages, denominated primary, secondary, and tertiary. Your attention is called to these three stages in the order mentioned.

The first or premonitory symptom manifests itself usually within twenty to thirty days after the subject has been inoculated, by the appearance of a slight abrasion at the point of infection; this abrasion is denominated a chancre, almost always single, sometimes multiple, when more than one, all appear simultaneously and disappear or heal in the same manner. The chancre usually disappears or heals within two or three weeks after its appearance; it is usually small, not larger than a turkey shot, sometimes elevated, sometimes flat, usually characterized by an indurated or hard base, having the appearaece of a disc lying loose in the superficial tissue, never suppurating, and exuding a colorless, transparent fluid, giving the ulcerated surface a varnished appearance. Its color is usually a brick red, sometimes a reddish gray, and, as a most important symptom in differential diagnosis, there is but slight pain elicited on pressure. This is the description given in our text books, which in the main we find to be a good one, but every case is more or less a law unto itself and every physician who has observed closely, coming in contact with syphilis in its primary stage, knows that there is no general rule that applies to all cases. I am slow to pronounce any erosion or abrasion of the genitals of recent manifestation, a syphalitic chancre; I have learned not to accept any primary lesion as absolutely pathognomonic; I usually reserve my diagnosis for the development of secondary symptoms.

We are taught that there is a period of from twenty to thirty days from the time of inoculation to the time of the appearance of the chancre; this has not always been the history given me of the cases I have treated. Three years ago in June past, a young man came to me with an abrasion of the penis located just back of the glans; the ulcer was small, about double the size of a pin's head, flat, soft, no indurated base, no resistance to touch. He told me that he had noticed the erosion only a few hours before coming to see me; that five days prior to this time he had had intercourse with a woman of questionable health; on being questioned about his opportunities for having chancres prior to this time of being infected, he assured me that he had had no chance for three and a half months before. Knowing the young man's reputation for truth and veracity, I had the utmost confidence in what he told me, and assured him that he had no syphilis. I prescribed a boracic acid powder for dusting the parts, recommended frequent bathing with a weak solution of peroxide of hydrogen, and a dressing of absorbent cotton to be applied after each washing. The erosion spread within the next two days to double its original size, but entirely healed within two weeks. Then followed a period of about six weeks of apparent immunity, when inagine my surprise at the appearance of squamous syphilides on the head, along the edge of the hair, around the scrotum and anus, and the brick dust or ham colored splotches in the palms of the hands and soles of the feet, with ulcers of mouth and tongue. I again questioned him about his previous chances of becoming infected before this particular time, as given me when first consulted; he reassured me that his first statement was entirely correct, and that he had had no other chance of catching the disease. I put him on a mercurial treatment and later followed it with iodide of potassium, which was kept up with short intermissions for two years, at the end of which time I pronounced him cured. This case I watched very closely; there was never any enlargement of the inguinal or other glands; there was no alopecia. A paronychia of some of the digits of both hands and feet was present, appearing along with the squamous syphilides, both of which disappeared within about a month, and again reappeared simultaneously within about thirteen months, but in a much milder form. This patient has shown no symptoms of a syphilitic nature now for the past sixteen months, having so far escaped all tertiary manifestations, I believe him cured. I relate this case in evidence that the period of incubation is not always twenty or thirty days; I have treated a number of patients for syphilis who have told me that the ulcer appeared within from seven to nine days after having been exposed. Now, for comparison, we will briefly mention the simple or soft chanchre. It is non-infecting, that is, it is not a constitutional malady. The chancroid rarely exists singly; often several appear simultaneously or successively by auto-inoculation; this, however, is not necessarily so, but in the soft chancre we no longer deal with simple excoriation, it is a true loss of substance. The skin is destroyed, the edges are perpendicular, as if punched out and slightly undermined; but let me stop to say that while this is the general rule, it is not always so. Sometimes the simple chancre is very shallow and also very small, and if you will treat your patient strictly aseptic—with strict cleanliness, and keep the glans well wrapped in borated cotton, frequently changing the dressing and frequently washing, you will have no succession of chancroids appearing on the parts. Then we cannot always tell whether we have a chancre or a chancroid to deal with; it is the ugly chancroid that quickest informs us of its simplicity; it is the indurated chancre that positively assures us that our patient has syphilis.

The chancroid is auto-inoculable, the chancre is non-auto-inoculable; you may have chancroid a hundred times, or as long as there is anything left for a chancroid to form on; you can have syphilis only once, and if you so will it, you can have that one case last you longer than your hundred cases of chancroid. With the chancroid you usually have a suppurating bubo; following the chancre you almost always have a bubo, but one that never suppurates. The mixed chancre is entitled to mention, but it would make my paper too long, so we will pass to the second stage.

As we have stated, the indurated, or syphilitic, bubo does not suppurate, neither do we have suppuration in secondary manifestations, or rarely so, and very slight when it does occur, the second period being a vegetative rather than a distinctive phase. The local accident has healed and from six weeks to two months have passed, the patient enjoying apparently complete immunity, when a roseola appears about the waist or upon the chest, small red spots, lenticular, with or without elevation, covered or not with epidermic scales, there exists a number of varieties but we will speak only of what ordinarily transpires. This roseola may be wanting, but most often is the most apparent symptom, and may be abundant or discrete; its color is of a delicate rose at the beginning, rapidly changing to a coppery red. They may be smooth and even with the cutaneous surface, though usually are elevated papules, scattered irregularly over the body, having the appearance of a concretion of epidermic tissue; at other times the roseola spreads en masse, or in irregular islets, then it is styled erythematous. This roseola may cover the entire trunk and limbs; it is rare on the face, but frequently is on the forehead along the edge of the hair; almost always the hair falls out in patches; the beard, the eyebrows and eyelashes sometimes fall out, but more rarely. In the mouth, on the lips, the internal surface of the cheeks, the tonsils, the palate and the tongue, are frequently found in greater or less abundance, ulcerated papular syphilides, surrounded by a bright red areola and covered with a whitish secretion. These mucus abrasions of the mouth and tongue are much more numerous and decidedly aggravated by the influence of alcohol and tobacco. The posterior cervical ganglia are implicated like those of the groin, but are equally indolent and never suppurate. About the anus and scrotum, on the penis and vulva are usually encountered papular or papulo-hypertrophic syphilides, also called mucus patches. These are further subdivided into ulcerative and hypertrophied syphilides; but, as what has already been said is amply sufficient to simplify and establish beyond question the diagnosis of secondary syphilis, I deem it expedient to call a halt and approach the tertiary period, in which the disease is no longer dangerous to any one save the syphilitic himself. Along the line of our march up to the third station the road for the patient has been all cauliflowers and roses, the danger existed only for others, and let me tell you, in my humble opinion, if we will treat our patient judiciously and intelligently, the patient himself faithfully carrying out our instructions, nineteen times out of twenty he will not be troubled with tertiary manifestations. But let the patient who is getting on so nicely abandon all internal treatment, believing himself cured, take to strong liquors and cigars, and before two months have passed everything will have returned in an aggravated form. It follows then that one must be temperate in all things, well nourished and avoid excesses of every kind. The third and last stage is directed to the destruction of the tissues of the body, as muscular, bone and glandular. The syphilitic virus itself is characterized in a general way by a proliferation of the connective tissue. This is especially why alcoholic beverages are harmful to syphilitics: drunkards being predisposed to a host of other maladies which owe their origin to proliferation with condensation of the connective cellular tissues, as cirrhosis of the liver, Bright's disease, chronic gastrititis, valvular insufficiency, a congested and œdematous capillary circulation, and a general malnutrition of the body brought about by continuous drinking, make a most fertile soil for tertiary ravages.

The most common tertiary lesions manifest themselves in the form of agglomerated tubercles deposited in the connective tissue, the so-called gummata. These accidents may show themselves any time after the third year, but usually do not appear until after the first few years of tertiary infection. They first appear as hard nodules, painful to the touch, then soften and break down exuding an ichorous liquid similar to a solution of gum; after this matter has escaped they heal by cicatrization. These gummata may develop in the brain, in the connective tissue of the glands, muscles, etc., and may give rise to various functional troubles too numerous to mention in this paper. Periosteal and bone lesions are the most important tertiary accidents. First we have an ostitis, attacking preferably the superficial bones. This ostitis may or may not result in caries or necrosis; we may have an exostosis or a hyperostosis or a destructive periostitis accompanied with osteocopic pains, nocturnal headaches, and every other kind of pain and ache that the human organism is heir to. I remember seeing a subject in the dissecting room wha had died with syphilis, the periosteum of the long bones and of the bones of the face and the head being almost entirely destroyed.

A few words on treatment and I have done. Different constitutions react very differently to the syphilitic poison. There are persons who receive very brief and very imperfect treatment, and who for years reveal no traces of the disease. On the other hand, there are persons who are carried through a most thorough and scientific course of treatment and for years reveal, off and on, ugly syphilitic lesions; but I think this only shows the susceptibility of the one for the virus and the power of resistance of the other. My observation has taught me that ugly secondary manifestations mean a comparatively ugly tertiary trouble, and that slight secondary symptoms mean, if properly treated, no tertiary lesions, notwithstanding some of our best authorities to the contrary. The strumous or cachectic subject is much harder to treat than the hearty and vigorous. Statistics show that syphilis is on the increase, but it appears to me that it is not so, at least in the South. Fifteen years ago I met with three times the number of cases that I meet with now, and I would like to appeal to the experience of every practitioner in Texas who has been in the practice for the last twenty years if this is not his experience. But I must get back to the treatment, which I promise to make short, for it can be told in a few words. Let me tell you that there is no disease that is so loathsome, so utterly repulsive, and yet at the same time so amenable to treatment as syphilis in the healthy, robust subject; but the great trouble is getting your patient to carry out your instructions. He gets weary of well doing too quick, you have got absolutely to scare him to death by telling him blood and thunder stories of the awful consequences of his disease, making him believe that if he fails to carry out your instructions fully and entirely he will die within a year and go where he feels like he ought to be, and every time he recovers from his fright you have got to scare him again and you have got to keep on scaring him for at least two or three years, perhaps longer, and the chances are if he is getting on nicely after awhile he gets to a point where you can't scare him much and he will neglect or entirely leave off treatment. And if he is not getting on nicely he will swear that you don't know anything about his case and quit you and fall into the hands of the other fellow, who can cure the worst case of "ral" a man ever had in six weeks, but who can't differentiate between an indurated bubo and an inguinal hernia, or papulo-squamous syphilides and simple eczema. We have but two specifics for syphilis, each one adapted to its particular stage of the disease, viz: mercury and iodide of potass. I always commence with my mercury on the appearance of the venereal sore, believing by so doing both the severity and duration of the second stage is very materially modified. I prefer for the first twelve months to use the ordinary blue powder, given three times a day in two to four grain does as tolerated by the patient. This should be continued without intermission for five or six months, then if the patient is doing well I leave the powder off for six weeks or two months and resume again with the same drug and continue four months longer; then again I leave off the treatment for two months and then put my patient on proto-iodide of mercury, one-fifth to one-third grain doses three times a day continuing this treatment for four months, then following this for the next four months with iodide potass., forty to sixty grains per day, then again my proto-iodide pellets for two months, when, in nineteen times out of twenty, my patient is cured if he has faithfully carried out my instructions. After two years have passed, if there has been no appearance of syphilitic taint for the past few months, I leave off the treatment and simply watch my patient the rest of his life. Supplementing internal treatment, I have my patient take hot baths frequently, looking well to the promotion of the excretory organs, and observing in the strictest sense possible the laws of hygiene. Do you know that going to the Hot Springs of Arkansas to get cured of your syphilis is all a mistake and a deception that enveigles the honest physician out of many a fee to which he is justly entitled and robs his patient unnecessarily out of many a hard earned dollar. The physicians of Texas, Louisiana, Mississippi, Tennessee, Missouri, and the territories contiguous to Arkansas are responsible for the education of the masses to the belief that these waters are a penacea for every syphilitic taint. You can treat your patient just as intelligently and with just as good results at home if he will faithfully do your bidding. The reason why, and the only reason, that your patient improves more rapidly there is because he carries out the instructions of Dr. Hobo, into whose hands he is there placed, to the letter, believing, inasmuch as he has gone to Hot Springs, he has to do or die, and he will always do. I know whereof I speak for I was born in Arkansas and lived there for twenty-five years, but never saw the famous watering place in my life. A visit to Hot Springs was always pathognomonic. I didn't go there for I didn't want to have people talking about me.

Physicians, eminent in their profession, are in a great many instances responsible for their failures in curing syphilis.

I have spoken of the difficulty of getting your patients to carry out your instructions, and now I am going to speak of some of the different methods used all over our land and country of administering mercury to the syphilitic patient, and I shall speak of them only to condemn them. We have the mercurial or vapor bath, which affords no little annoyance and inconvenience to the patient; we have practiced the hypodermic injection of the bichloride, upon which the patient looks with deadly horror from one sitting to another, and during the sittings he wishes he had never been born, to say nothing of the chances it subjects him to of having subcutaneous suppurating abscesses; then we have the rubbing-in process, the so-called inunction of mercurial ointment, which makes your patient feel like he is not fit to associate with white folks, but ought to go off and bed up with the hogs and die. In the name of heaven isn't your patient nasty enough with his chancres and his pustules, with his foul breath and his putrid sore mouth, without besmirching him with this dirty, filthy, loathsome ointment? No doubt, gentlemen, but all of you have practiced some one or all of these methods of treatment, but for heaven's sake quit it. I don't say that the means are ineffectual, for it is effectual in two ways—if you can hold your patient it is effectual in mercuralizing him, but it is most usually effectual in running him off before you get him cured. If you want to mercuralize your patient, you can do it as promptly and as efficiently by internal administration as you can by any other method. It may be possible that now and then you meet with a case where internal administration produces gastro-intestinal disturbances, but this can be overcome by combining with your mercury a little opium. This simplifies your treatment, your patient always has his medicine where he can lay his hand on it, it is both palatable and clean, he is not in constant dread of the hour that shall roll around for him to be dosed, he is more apt to carry out your instructions, and your results will be just as good as if you adopt and carry out any other mode of administration.

I believe that occupation has much to do with eliminating the syphilitic virus. Several years ago two young men of my acquaintance were away from home over night on a jaunt; they each had sexual congress with the same woman, who was syphilitic; they both had chancres appearing simultaneously as a result twenty-six days afterward. One of them consulted me in the forenoon and the other in the afternoon of the same day. One was a clerk in a dry goods store, the other lived an active, outdoor life. They were both hearty, robust young men. I put them both on the same treatment, and the same line of treatment was carried out with both of them, but the clerk, who led a sedentary life, had a much severer case than the other fellow, and I have noticed that syphilitic patients confined to indoor work, as a rule, have a harder time of getting cured than those living an active, outdoor life. It holds to reason that active, outdoor labor, promoting free perspiration, stimulating the excretory system, thereby facilitating the elimination of the specific poison, that the attack will be of shorter duration, that the symptoms will be much milder in their course, everything else equal, than in the patient who leads a sedentary life.

